# Circular polarized light-dependent anomalous photovoltaic effect from achiral hybrid perovskites

**DOI:** 10.1038/s41467-022-35441-9

**Published:** 2022-12-13

**Authors:** Tingting Zhu, Jie Bie, Chengmin Ji, Xinyuan Zhang, Lina Li, Xitao Liu, Xiao-Ying Huang, Wei Fa, Shuang Chen, Junhua Luo

**Affiliations:** 1 State Key Laboratory of Structural Chemistry, Fujian Institute of Research on the Structure of Matter, Chinese Academy of Sciences Fuzhou, 350002 Fujian, China; 2 School of Physical Science and Technology, ShanghaiTech University, 201210 Shanghai, China; 3 School of Chemistry and Chemical Engineering, Jiangxi Normal University, 330022 Nanchang, China; 4 University of Chinese Academy of Sciences, 100049 Beijing, China; 5 Fujian Science & Technology Innovation Laboratory for Optoelectric Information of China, Fuzhou, 350108 Fujian, P. R. China; 6 Kuang Yaming Honors School, Nanjing University, 210023 Nanjing, Jiangsu China; 7 National Laboratory of Solid State Microstructures and Department of Physics, Nanjing University, 210093 Nanjing, Jiangsu China; 8 Institute for Brain Sciences, Nanjing University, 210023 Nanjing, Jiangsu China

**Keywords:** Optical materials, Photonic crystals

## Abstract

Circular polarized light-dependent anomalous bulk photovoltaic effect - a steady anomalous photovoltaic current can be manipulated by changing the light helicity, is an increasingly interesting topic in contexts ranging from physics to chemistry. Herein, circular polarized light-dependent anomalous bulk photovoltaic effect is presented in achiral hybrid perovskites, (4-AMP)BiI_5_ (**ABI**, 4-AMP is 4-(aminomethyl)piperidinium), breaking conventional realization that it can only happen in chiral species. Achiral hybrid perovskite **ABI** crystallizes in chiroptical-active asymmetric point group *m* (*C*_*s*_), showing an anomalous bulk photovoltaic effect with giant photovoltage of 25 V, as well as strong circular polarized light - sensitive properties. Significantly, conspicuous circular polarized light-dependent anomalous bulk photovoltaic effect is reflected in the large degree of dependence of anomalous bulk photovoltaic effect on left-and right-CPL helicity, which is associated with left and right-handed screw optical axes of **ABI**. Such degree of dependence is demonstrated by a large asymmetry factor of 0.24, which almost falls around the highest value of hybrid perovskites. These unprecedented results may provide a perspective to develop opto-spintronic functionalities in hybrid perovskites.

## Abstract (web summary)

The manipulation of anomalous photovoltaic current by changing the light helicity has attracted an increasing interest in the field. Here, the authors realize circular polarized light-dependent anomalous bulk photovoltaic effect in achiral hybrid perovskites with a large asymmetry factor of 0.24.

## Introduction

Anomalous photovoltaic effect (APE)-under continuous uniform illumination, a steady photocurrent and above-bandgap photovoltage can be generated in asymmetric single-phase compounds, has been attracting increased interest in photoelectronic and photovoltaic field^1–3^. In general, photovoltaic effect can be manipulated by changing attributes of the incident lights because light will affect the separation of photogenerated holes and electrons for materials with broken inversion symmetry^4^. In this context, photovoltaic effect can be resolved into two parts, linear and circular^5^. Of particular interest is circular polarization light-dependent bulk photovoltaic effect (CBPE), which is a second-order nonlinear photoelectric response. A steady photovoltaic current exhibits a circular polarization angle dependence under the excitation of circularly polarized light (CPL)^6–8^. Such effect is closely associated with asymmetric structures that affect spin and bulk photovoltage as well as CD absorption differences that affect CPL-matter interactions^9,10^. Take the typical asymmetric BiFeO_3_ as an example, its ferroelectric domain arrangements exhibit obvious CBPE, which roots in differential interactions associated with differential circular dichroic (CD) behaviors between CPL and domain variants^11^. However, analogs of this inorganic material with CBPE require complicated fabrication technics with high temperature process^12,13^. Therefore, it is of significance to explore economically friendly materials for develop CBPE.

**Fig. 1:**
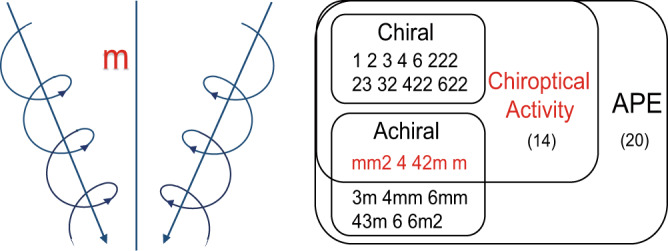
Analysis related to point group. Chiroptical activity from an entity with *m* point group (Left); Relationship between point group, chiroptical activity and APE (Right).

Solution-processed organic-inorganic hybrid perovskites are promising due to their low cost, structural flexibility, and excellent semiconducting properties^14,15^. Nevertheless, their development in CBPE is usually hindered by time-consuming chiral components which is essential for inducing the unique chiroptical activities (e.g. CD) and asymmetry^16,17^. Although great efforts on exploring CBPE in chiral hybrid perovskite have been made, and only a few cases exhibit CBPE, such as, [(R)-(MBA)]_2_PbI_4_^18^, [(R)-β-MPA]_2_MAPb_2_I_7_^19^, [(R)-β-MPA]_4_AgBiI_8_^20^. In fact, without chiral components, same essential factors such as chiroptical activities can also be achieved in achiral crystals with four asymmetric point groups (*m*, *mm*2, 4 and 42 *m*)^21–24^. For example, for asymmetric *m* point group, one may imagine two forms of ‘vortices’ for the orientation of optical axes, of which the rotation along one of the axes is the right-hand screw, while along the other axis obtained by the mirror plane symmetry operation is the left-hand screw (Fig. 1, left). Therefore, such achiral crystals also can generate positive and negative chiroptical signals subject to different directions of the incident light^25,26^. Moreover, apart from chiroptical activities, these four asymmetric achiral point groups can also offer great opportunities for generating APE, which spontaneously drives the separation and transport of photoexcited carriers thus providing powerful support for CBPE (Fig. 1, right)^27^. However, the number of crystals crystallizing in the above point group is scarce especially in the organic-inorganic systems. To date, realizing CBPE in achiral hybrid perovskites has not been reported.

Here, by using low-cost achiral organic amines, we report CBPE in achiral hybrid perovskites (4-AMP)BiI_5_ (**ABI**, 4-AMP is 4-(aminomethyl)piperidinium). Achiral hybrid perovskite **ABI** crystallizes in the chiroptical-active asymmetric point structure *Cc*, enabling **ABI** to be CPL-sensitive due to its right and left-handed screw optical axes. Meanwhile, such a chiroptical-active asymmetry structure arouses a superior APE with steady-state photovoltage (*V*_oc_) of about 25 V. Importantly, coupled with the strong CPL-matter interaction, these advantageous factors enable **ABI** to exhibit CBPE. This conspicuous effect is reflected in the large degree of dependence of anomalous photovoltaics on left-and right-CPL helicity, which is demonstrated by a large asymmetry factor of 0.24.

## Results

### Crystal structures

**Fig. 2:**
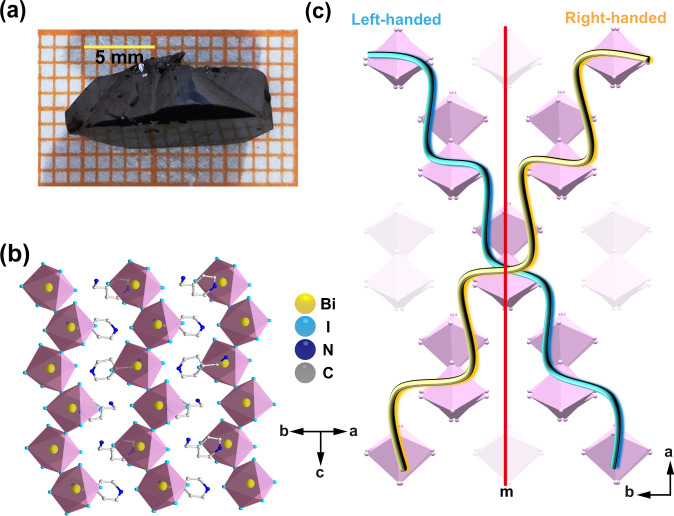
Structural characteristics of ABI. **a** Bulk single crystals of **ABI**. **b** The zig-zag chains structure of **ABI**. **c** The relationship between the left-handed helix and right-handed helix relative to the m symmetry plane in **ABI** crystals.

Without use of any chiral organic amines, black bulk single crystals of **ABI** with sizes up to 15 × 6 × 5 mm^3^ are grown via simple and low-cost solution cooling process. (Fig. 2a), and the power X-ray diffraction peaks validate their phase purity (Supplementary Fig. 1). Single crystal X-ray diffraction reveals that **ABI** adopts a non-centrosymmetric space group *Cc* as verified by the second harmonic generation (Supplementary Table 1 and Supplementary Fig. 2). Notably, space group *Cc* belongs to 4 chiroptical-active point groups (*m*, *mm*2, 4 and 42 *m*). As illustrated in Fig. 2b, **ABI** displays the structure of alternating arrangement between zig-zag chains and discrete 4-AMP cations. It is worth noting that the strong N-H┅I hydrogen bonds between the N and I atoms well hold inorganic frameworks in the crystal lattice, and organic ammonium cations regularly lie between the inorganic chains (Supplementary Fig. 3). The interaction asymmetric hydrogen-bonding between the inorganic BiI_6_ octahedral chains and 4-AMP cations leads to helical distortions in the whole chiroptical-polar structure (Supplementary Tables 2–3). This polar structure produces an electric polarization (***P***_***m***_) along the *ac* plane. The *P*_*m*_ value along the *a*- and *c*-axis are evaluated to be 0.61 μC cm^−2^ and 0.45 μC cm^−2^, respectively, by point charge model calculations (Supplementary Fig. 26 and Supplementary Table 5). Moreover, combining Figs. 1 and 2c, right-handed and left-handed helixes could be found in **ABI** by connecting the nearest neighbor BiI_6_ octahedral skeletons. Such helixes of opposite handedness can transform into each other by a m slide surface and are simultaneously distributed in a crystal structure **ABI** at a ratio of 1:1 (Supplementary Fig. 4). Besides, the existence of chiroptical activity in the **ABI** isn’t depend on the intrinsic characteristics of the racemic units but is solely determined by its symmetry^21,24^. Hence, non-centrosymmetric stacking in **ABI** is responsible for chiroptical-active signals, which will facilitate to demonstrate the realization of CBPE.

### Optical properties

**Fig. 3:**
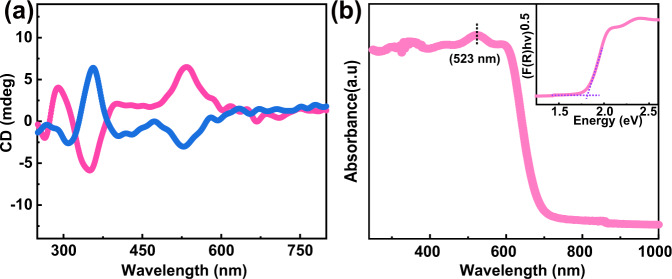
Optical characterizations of ABI. **a** Circular dichroism spectra of **ABI** crystals by using integrating sphere to detect the diffuse reflectance. **b** Absorption spectrum and inset shows the corresponding Tauc plot. Source data are provided as a Source Data file.

The circular dichroism (CD) measurement is a powerful approach to support chiroptical activity. In a general solid-state CD measurement, a piece of **ABI** single crystal is used to mix and ground with a certain proportion of KBr power. It can be seen obvious opposite positive and negative CD signs at the same wavelengths from Supplementary Fig. 5. However, it is difficult to discuss the CD properties of this sample at this weak signal intensity. In this regard, The CD measurements by using microcrystals are further carried out as shown in Fig. 3a. obviously, the CD signals appear to be at almost the same wavelengths (290, 356, 442, 523 nm) but with opposite signs. The bisignated CD signals (523 nm) correspond to the first excitonic transition band edge (523 nm), which are consistent with the absorption spectrum (Fig. 3b). In addition, to confirm the reliability of the CD measurements, we also use thin film samples to test CD (Supplementary Figs. 6 and 7). Corresponding angular dependence CD spectra can verify that the chiroptical activity of ABI is associated with the directionality of incident light. This phenomenon is consistent with the chiroptical activity characteristics of *m* point group. In addition to CD absorption, the corresponding optical absorption of **ABI** are evaluated. As shown in Fig. 3b, the absorption cut-off of **ABI** locates at 680 nm, and the estimated bandgap is 1.81 eV. This bandgap is much narrower than those of classic compounds with APE, such as BiFeO_3_ (~2.7 eV)^28^, BiVO_4_ (~2.43 eV)^29^, and (3-pyrrolinium)CdCl_3_ (~3.5 eV)^30^. Such a narrow bandgap shows that **ABI** can absorb the visible-light illumination to facilitate the generation of carriers, thereby realizing the broadband device applications. Meanwhile, we estimate the electronic band structure and density of states of **ABI** by using the first-principles calculations both without and with consideration of spin-orbit coupling (SOC) effect of the heavy atoms within this perovskite (Supplementary Figs. 8 and 9). Remarkably, after considering the SOC effect, I-5p states mainly attribute the valence band while both Bi-6p states and I-5p states dominate the conduction band. That is, heavy atoms Bi and I of **ABI** are the domain contribution of SOC effect, which will lead to generate the circular photoexcitation^31,32^. Consequently, the optical bandgap of **ABI** relies on the inorganic BiI_6_ framework, which is similar to that of 2D hybrid organic-inorganic perovskites^33–35^.

### Anomalous bulk photovoltaic effect

**Fig. 4:**
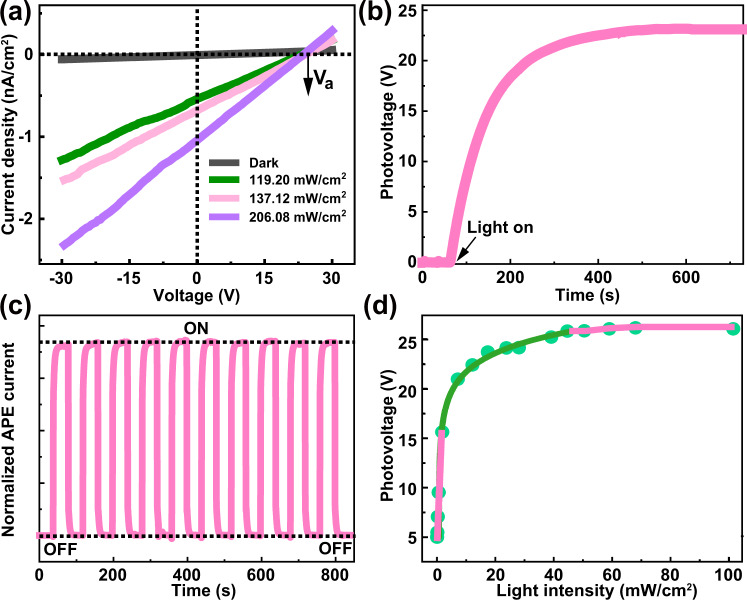
Anomalous photovoltaic effect (APE) behaviors of ABI. **a** Current-voltage (*I*–*V)* traces under different light intensities (*I*_t_ > 89 mW/cm^2^). **b** Open-circuit voltage (*V*_a_) versus time when the light is turned on. **c** The real time-dependent photocurrent of device under 405 nm light illumination under 0 V bias. **d** Photovoltages versus light intensity. Experimental results (green) and photovoltage model (pink and dark green). Source data are provided as a Source Data file.

Good semiconducting performance needs to be evaluated after having the evidence for chiroptical activity. Subsequently, the photoelectric characteristics are measured on surface of **ABI** bulk single crystals along three directions (*a*-, *b*-, *c*-axes) under a visible 405 nm laser irradiation (Supplementary Fig. 10). As given in Fig. 4a, for the electrodes aligning in the *a*-axis, a short-circuit current density *J*_sc_ of 0.68 nA/cm^2^ is observed under 119.2 mW/cm^2^ illumination. Emphatically, the obtained open-circuit voltage *V*_*a*_ is up to 25 V, which is ~13 times larger than its bandgap, revealing an obvious APE along the *a*-axis. Such an enormous voltage is much higher than that of the well-known inorganic ferroelectric BiFeO_3_ and is comparable to that of wide-band hybrid semiconductor (3-pyrrolinium)CdCl_3_^30^_._ Meanwhile, the photovoltaic current and photovoltage are repeatable under on/off switching, verifying the favorable APE stability of **ABI** (Fig. 4b, c). Admittedly, the time required for the open circuit voltage to reach saturate under the illumination is relatively long. In-depth understanding find that drift of ion/vacancies should be one of the important influencing factors. Owing to the weak Bi-I bonds and shorter distance to the nearest I^−^ vacancy, I^−^ may be the main migrating species in **ABI**^36,37^. The resulting diffusion current delays the process of carrier separation and transition from non-equilibrium to equilibrium, which eventually takes a long time to reach equilibrium. That is, hysteretic photovoltage saturation may derive from the light-induced self-poling process^38^. Moreover, superior photovoltage exists over a wide wavelength ranging from 266 nm to 637 nm (Supplementary Figs. 11–14), which is well in accord with the wide band absorbance of **ABI**. Particularly, the photovoltages of **ABI** varied with light intensity is observed in Fig. 4d. In detail, under low light intensities, photovoltage is linearly proportional to the incident light intensity; when the light intensity increases to 21 mW/cm^2^, it becomes a weak logarithmic dependence *V*_oc_ ∝ ln *I*_*t*_ (*I*_t_ is the light intensity at the illuminated surface); when *I*_t_ increases by 89 mW/cm^2^, *V*_oc_ tends to be saturated. This behavior is well in agreement with that of most ferroelectric and polar materials, such as LiNbO_3_: Fe^39^, LiNbO_3_^2^, and PSI crystals^3^. Besides, the dark current density is as low as 6.7 pA/cm^2^ (*V*_bias_ = 0 V). Such a low dark current significantly enables a considerable on/off ratio of current up to 200, critical for the noise performance of photodetector. As a result, the detectivity is estimated to be 4 × 10^6^ Jones. The above results endow **ABI** a great potential for achieving conspicuous CBPE.

To study the origin of APE in chiroptical-active achiral hybrid perovskite **ABI**, we discuss the correlation between polarity and APE. As depicted in Supplementary Fig. 15a, prominent *V*_*c*_ (=5 V) is observed for the electrodes aligning in the *c*-axis. However, for the electrodes aligning *b*-axis, almost negligible *V*_*b*_ signals could be detected. Thus, APE only exists in the direction parallel to the *ac* plane. This phenomenon is well consistent with that of the difference of *P*_m_ in different axial directions (0.61, 0.45, 0 μC/cm^2^ along the *a*, *c, b*-axis respectively), indicating there is a close relationship between *P*_m_ and APE. Specifically, the displacement of infinite negative charge center [BiI_5_]^2−^ and the positive charge of (4-AMP)^2+^ together generate a polarization on the *ac* plane. Then, the illumination leads to photo-induced electrons and holes to separate toward opposite directions along the *ac* plane, thereby inducing an electric current and photovoltage (Supplementary Fig. 15b). In such a case, with the saturation of polarization and pyrocharge, the photovoltage reaches a maximum, which is due to the localization of non-equilibrium carriers in relatively deep traps^40–42^. Such APE containing this *P*_*m*_ can be explained by the shift current model as reported in BiFeO_3_^41^, LiNbO_3_^42^, (K, Ba) (Ni, Nb) O_3 – δ_^43^, TTF-CA^44^.

### Circular polarized light-dependent anomalous bulk photovoltaic effect

**Fig. 5:**
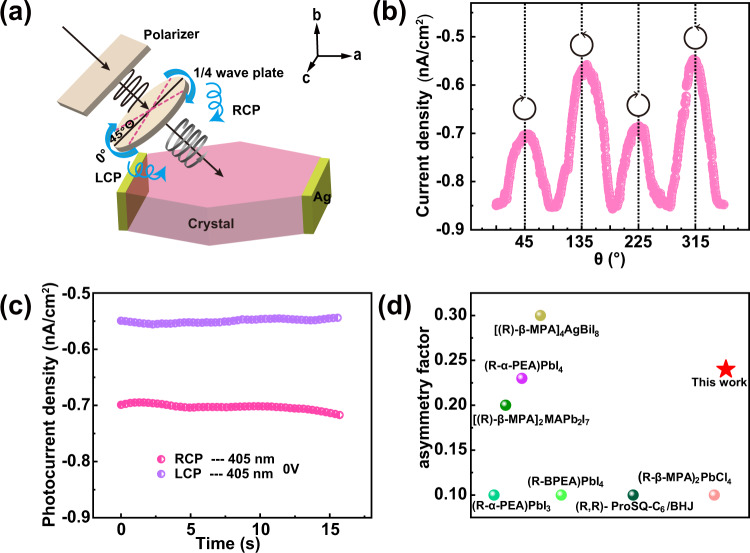
Circular polarization light-dependent bulk photovoltaic effect (CBPE) in ABI. **a** The schematic view of the testing the CBPE device. **b** Photocurrent density versus the linear polarization angle as tuned by the λ/4 plate under the illumination of 119.20 mW/cm^2^. Black arrows indicate right- and left-handed circularly polarized light (RCP, LCP). **c** The photocurrent density as a function of time under LCP-405 and RCP-405 nm and the irradiation of 119.20 mW/cm^2^. **d** Experimental asymmetry factors of some reported devices with CBPE. Source data are provided as a Source Data file.

The combination of above the chiroptical activities and good semiconducting features could provide great opportunities to achieve CBPE based on **ABI**. Firstly, we performed CPL photoelectric testing on **ABI** bulk crystals and the direction of electrodes is along the *a*-axis. The incident CPL is controlled by a polarizer and a quarter-wave plate and the CPL intersects the *ac* plane (Fig. 5a). The following experiments are all carried out under zero bias. Interestingly, there is a strong CBPE - a steady anomalous photovoltaic current has an obvious dependency on light helicity (Fig. 5b and Supplementary Fig. 16). Notably, photocurrent achieved under RCP illumination is obviously higher than that of under LCP illumination with the identical intensity regardless of the wavelengths of 266, 405, and 520 nm (Fig. 5c and Supplementary Figs. 17–18). These results demonstrate crystals of **ABI** have good distinction capacity between RCP and LCP photons. We speculate that this ability is most likely attributable to the higher free carrier generation efficiency under RCP. Specifically, **ABI** absorbs more right circular photoexcitation and generates more electron-hole pairs, thus more spin-triplets are generated due to the enhanced spin flipping process through SOC^31,32^. Furthermore, this ability is evaluated based on the anisotropy factor of photocurrent, *g*_Iph_, which is calculated as *g*_Iph_ = 2(*I*_R_-*I*_L_)/(*I*_R_ + *I*_L_). ${I}_{ph}^{R}$ and ${I}_{ph}^{L}$ are the photocurrent under LCP and RCP light illumination respectively. The *g*_Iph_ value is determined to be 0.24 under the 405 nm laser illumination. Moreover, such CBPE can exist over a wide wavelength ranging from 266 nm to 637 nm (Supplementary Fig. 19), which is well in accord with the wide band absorbance of **ABI**. It is important to realize CBPE in achiral hybrid materials and obtain a large *g*_Iph_ of 0.24. This value is superior to most chiral organo-inorganic hybrids, such as chiral perovskites, [(R)-β-MPA]_2_MAPb_2_I_7_ single crystal devices and (α-PEA)PbI_3_ films devices^19,32^. Experimental *g*_Iph_ of some reported devices with CBPE are summarized in the Fig. 5d and supporting information (Supplementary Table 4). Further, *g*_Iph_ of **ABI** at *V*_bias_ = 10 V is evaluated to be about 0.024, less than the polarization ration driven by APE, suggesting that large anisotropy factor is not only derived from the chiroptical activity of the structure, but also the intrinsic photovoltaic characteristics. Therefore, we conclude that the APE serves to separate electron-hole pairs, thus giving rise to enhanced CPL response and distinguish ability.

When the direction of incident light is reversed to that of Fig. 5a (Supplementary Fig. 20), the CPL-helicity dependence of the photocurrent switches between for left and right CPL (Supplementary Fig. 21). These results indicate that the optical axes are intersectant to *ac* plane, which is in line with the right- and left-handed helixes in structure of **ABI**. Besides, we monitor the stability of **ABI** bulk crystals, including XRD pattern (Supplementary Fig. 22), thermal stability (Supplementary Fig. 23), *V*_oc_ (Supplementary Fig. 24), and CPL detection (Supplementary Fig. 25). Corresponding original electrodes and single crystals are stored in the ambient environment without any encapsulation for 1 month to check. The results reveal negligible degradation of **ABI** under the ambient storage. Such good stability of **ABI** can compare to that of low-dimensional hybrid lead-halide layered perovskites, which is conducive to practical device application^32^.

## Discussion

In conclusion, CBPE has been successfully presented in achiral hybrid perovskites **ABI** whose chiroptical activities are demonstrated by a pair of CD signals with opposite signs, and a robust CPL response. Meanwhile, the intrinsic asymmetric chiroptical-active structure of **ABI** arouses a superior APE with *V*_oc_ of about 25 V. Remarkably, both chiroptical activity and APE will serve CBPE. This conspicuous effect is reflected in the large degree of dependence of anomalous photovoltaics on left and right optical rotation, which are demonstrated by a large asymmetry factor (0.24). These exciting results paved an avenue to develop emergent spintronics and related applications.

## Methods

### Synthesis and crystal growth

Bi_2_O_3_ (0.35 g) is dissolved in HI solution (45%, 20 ml). Thereafter, 4-(aminomethyl)pyridinium (10.0 mmol, 1.08 g) is added dropwise. The mixture is stirred and heated to dark red clear solution. 24 h later, large dark-red crystals of **ABI** are obtained by slowly cooling the solution (Supplementary Fig. 1).

### Single crystal structure determination

Single-crystal X-ray diffractions for **ABI** are performed on a Bruker APEX-II diffractometer with the Mo Kα radiation at 209 K and 280 K. The APX3 software is used for data reduction. The structures of **ABI** are solved by direct methods and then refined by the full-matrix least-squares refinements on *F*^2^ using SHELXLTL software package. Crystal data and structural information for **ABI** have been listed in Tables S1-S3, and CCDC 2087603 contain the crystallographic data for **ABI** in this paper.

### Physical properties measurements

Powder X-ray diffraction (PXRD) of **ABI** is recorded on a Rigaku MiniFlex diffractometer at room temperature. The diffraction patterns are collected in the 2*θ* range of 5°−50° with a step size of 0.02°. The experimental PXRD patterns match fairly well with the simulated data based on the single-crystal structure, which confirm the pure phase of **ABI** (Supplementary Fig. 1). TGA is conducted on a Netzsch STA449C thermal analyser in the temperature range of 310–1200 K and recorded at heating rate of 10 K min ^−1^. These samples remain thermally stable up to 523 K (Supplementary Fig. 23). SHG experiments of **ABI** are performed on powder samples using a Nd:YAG laser (*λ* = 1064 nm, 5 ns pulse duration, 1.6 MW peak power, 10 Hz repetition rate) (Supplementary Fig. 2). The UV absorptions in the solid-state are measured at room temperature on a PE Lambda 950 UV-Visible spectrophotometer with BaSO_4_ used as 100% reflectance reference. The optical bandgap is obtained from the Tauc equation: [*hνF*(*R*_∞_)]^0.5^ = *A*(*hν*-E_g_). Where *h* is the Planck’s constant, *ν* is the frequency of vibration, *A* is the proportional constant, *E*_*g*_ is the optical band gap, *F*(*R*∞) is the Kubelka-Munk function: *F*(*R*_∞_) = (1−*R*_∞_)^0.5^⁄ 2*R*_∞_ (Fig. 3b). Single-crystal structure data of **ABI** is used for the theoretical calculations.

### Computational details

Band structure and partial density of states (PDOS) are performed by the first-principles calculations, which are implemented using the Vienna ab initio simulation package (VASP). The Perdew-Burke-Ernzenhof (PBE) exchange-correlation functional is employed within a generalized gradient approximation (GGA)^45^. The van der Waals interactions are described using the empirical correction in the Grimme scheme (D3)^46^. For electronion interactions, an energy cut-off of 520 eV is implemented with the projector-augmented wave (PAW) potentials^47^. The Gaussian smearing with the width of 0.05 eV is used. The convergence tolerance of the total energy is 10^−7^ eV and the residual forces on all atoms are less than 0.02 eV/Å. The Brillouin zone is sampled with a Monkhorst−Pack *k*-point mesh with a grid spacing of about 2π × 0.01 Å^−1^. For heavy Bi atoms and I atoms, the spin-orbital coupling effects are considered.

### Photovoltage measurements

The current versus voltage (*I*–*V*) and photocurrent versus time (*I*–*t*) measurements are performed by using a Keithley 6517B source meter on the planar-type devices at room temperature. Different incident power incident light intensities are measured by light power meter. *I*–*V* tests under the 266, 377, 405, 520, and 637 nm continuous-wave lasers are collected. Single-crystal devices of **ABI** for all the photoelectric measurements are fabricated using millimeter crystals that appeared in Fig. 2a. The surface is cleaned under nitrogen flow before device fabrication. Silver electrodes are coating on the two sides of top wafers, and the channels between neighboring electrodes along the *a, b, c*-axes have areas of 0.03, 0.051, and 0.028 cm^2^, respectively. CPL measurements are applied based on a linear polarizer and a quarter-wave plate on crystal **ABI**. The pump is right-handed circularly polarized when the rotation angles are 45° and 225°, and is left-handed circularly polarized when the rotation angles are 135° and 315°, respectively. The detectivity (D*) is calculated through

1 $${D}^{\ast }=\frac{{I}_{ph}/{A}_{device}}{\left(\left({P}_{in}/{A}_{laser}\right)\times {\left(2e^{{I}_{dark}}/{A}_{device}\right)}^{0.5}\right)}$$

### Solid-state circular dichroism measurements

Solid-state circular dichroism (CD) measurements are measured on Bio-Logic MOS450 circular dichroism spectrophotometer and Chirascan V100 CD spectrometer (Applied Photophysics, UK0, with a solid sample intergrating sphere apparatus IS.3.), respectivily.

### Powders

KBr pellets are used as the matrixes, the scanning rate is 500 nm min^−1^ and the data interval is 1 nm. A piece of bulk crystal of **ABI** is randomly selected and mixed with the appropriate weight of KBr and fully ground to get powder samples. 50 mg of each mixed powder sample is pressed to uniform and transparent pellets for CD tests.

### Film

The films of **ABI** are fabricated by spin coating method with quartz glass as substrate. Substrates with desired dimension are cleaned in an ultrasonic cleaner using detergent, deionized water, ethanol, and acetone in sequence for 30 minutes of each. Next, the substrate surface is cleaned by ultraviolet-ozone cleaner for 20 minutes. The precursor solutions for the **ABI** are prepared by dissolving the **ABI** microcrystals in DMF (0.56 mol/L) for CD measurements. To form the films, 60 μL of the precursor solution is spread on the cleaned surface of the substrate, and then spun at 1000 rpm for 15 s, 3000 rpm for 25 s. Finally, the as-fabricated films are annealed at 80 °C for 15 minutes on a hot-plate to induce crystallization. The resulting film size is ~2 × 2 × (1 × 10^−4^) cm^3^.

## Supplementary information

Supplementary Information

## Data Availability

All relevant data are presented via this publication and Supplementary Information. The X-ray crystallographic coordinates for structures reported in this study have been deposited at the Cambridge Crystallographic Data Centre (CCDC), under deposition numbers: CCDC 2087603. These data can be obtained free of charge from The Cambridge Crystallographic Data Centre via www.ccdc.cam.ac.uk/ get structures. The authors declare that all data supplementary to the findings of this study are available within the paper. Source data are provided with this paper.

## Source data

Source Data
