# Supplementary material for: Circular polarized light-dependent anomalous photovoltaic effect from achiral hybrid perovskites
Source: Nat Commun. 2022 Dec 13;13:7702. doi: 10.1038/s41467-022-35441-9 (PMC9747807; doi:10.1038/s41467-022-35441-9)
Supplement: Supplementary file 1 — Supplementary Information [file 41467_2022_35441_MOESM1_ESM.pdf]

Supplementary information

Circular polarized light-dependent anomalous photovoltaic effect from achiral hybrid perovskites

Tingting Zhu et al.

Supplementary Figures

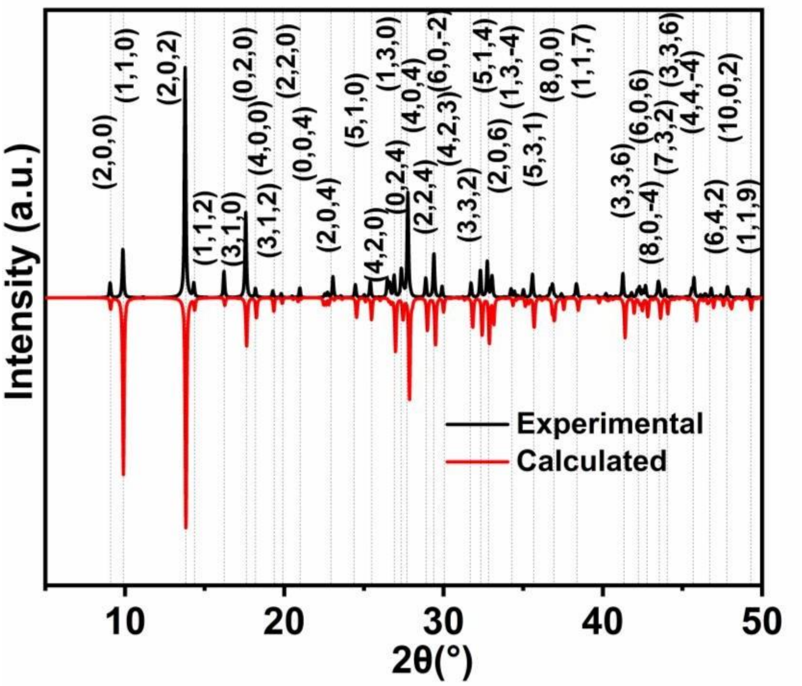

Supplementary Figure 1. Experimental and simulated powder X-ray diffraction patterns for **ABI** and labeled diffraction peaks.

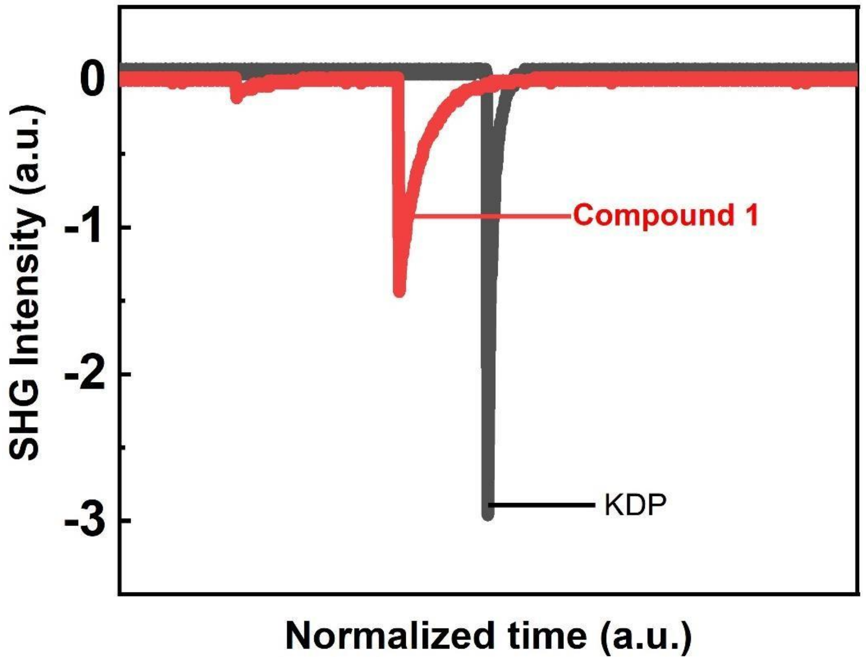

Supplementary Figure 2. Oscilloscope traces of SHG signals of **ABI.**

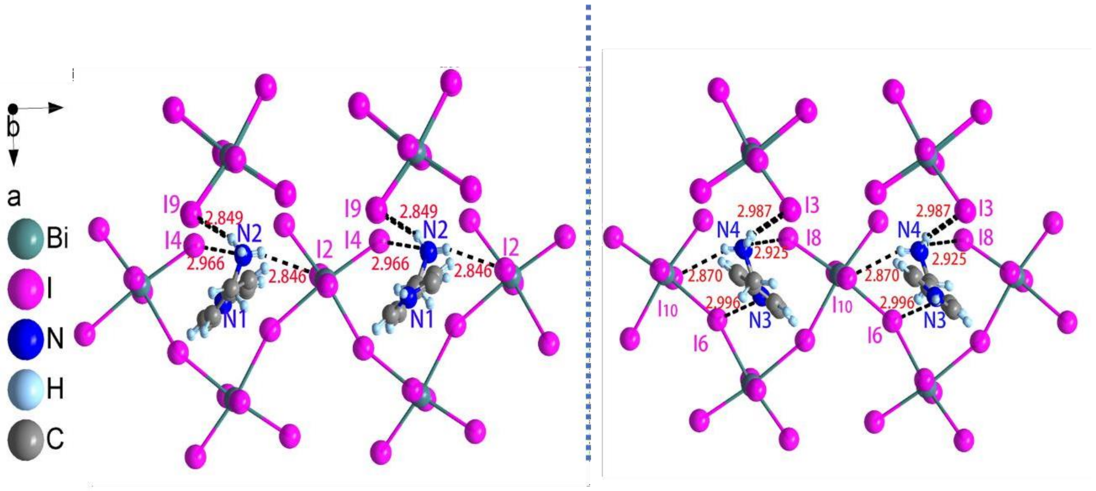

Supplementary Figure 3. N-H · I hydrogen bond configurations of **ABI.**

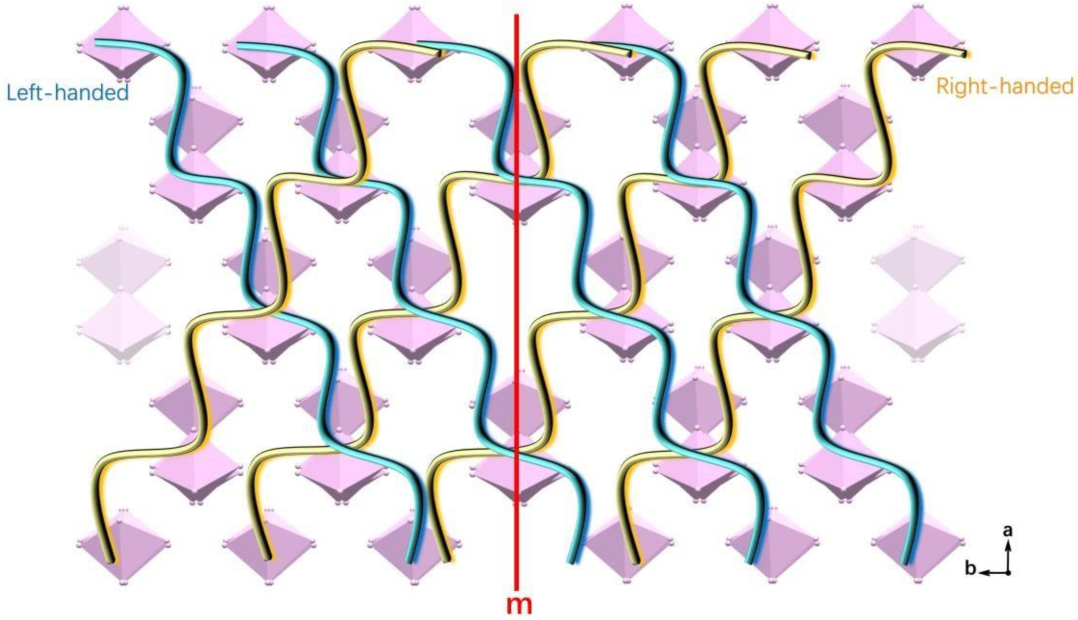

Supplementary Figure 4. The packing and arrangement of left-handed and right -handed helix relative to the m symmetry plane in **ABI** crystals.

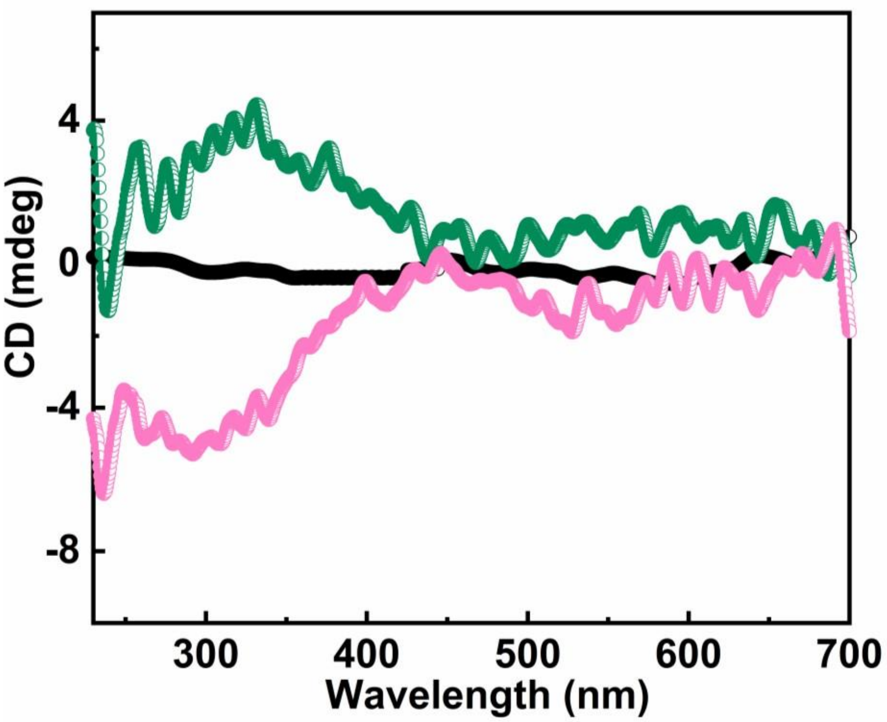

Supplementary Figure 5. Solid-state circular dichroism spectra using the mixed **ABI** and KBr powder sample.

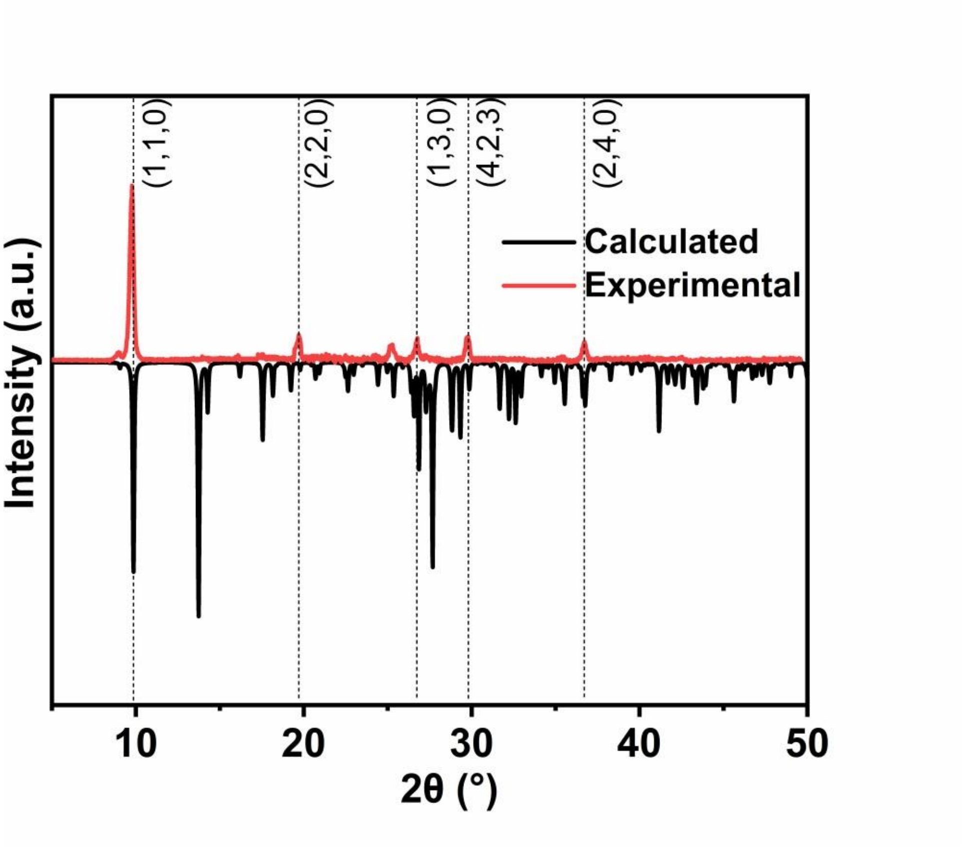

Supplementary Figure 6. Experimental and simulated X-ray diffraction patterns of **ABI** films.

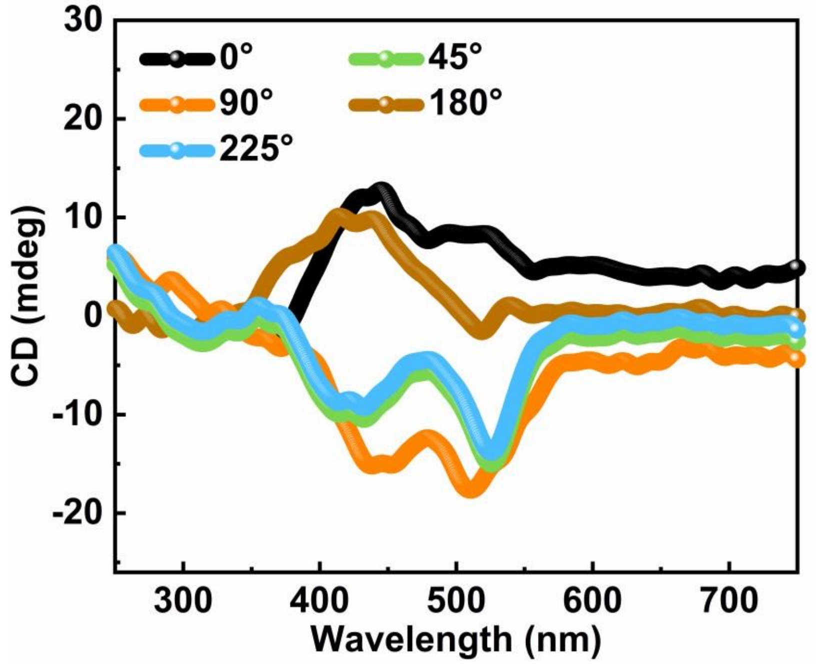

Supplementary Figure 7. Circular dichroism spectra of **ABI** films with

different rotation angles.

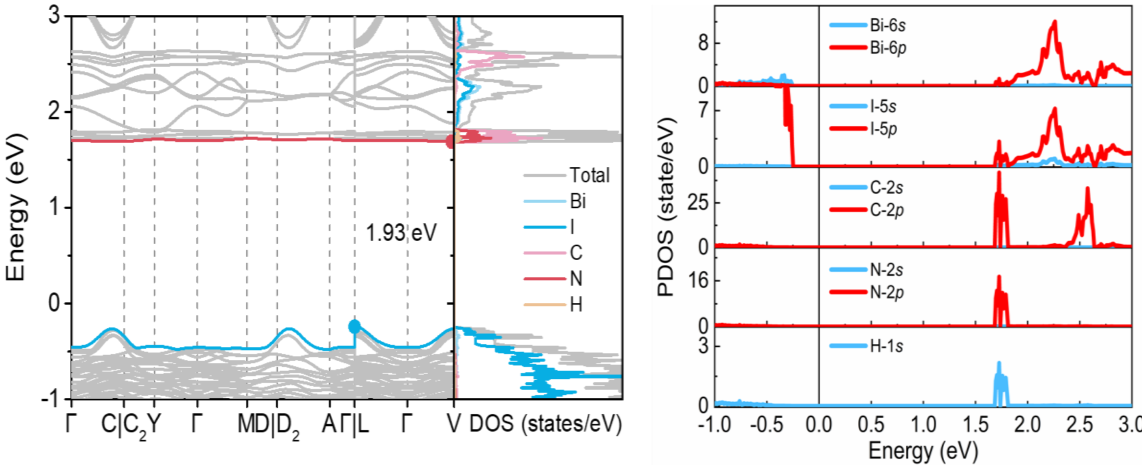

Supplementary Figure 8. Calculated band structure and Partial density of states spectra of **ABI** using PBE-D3.

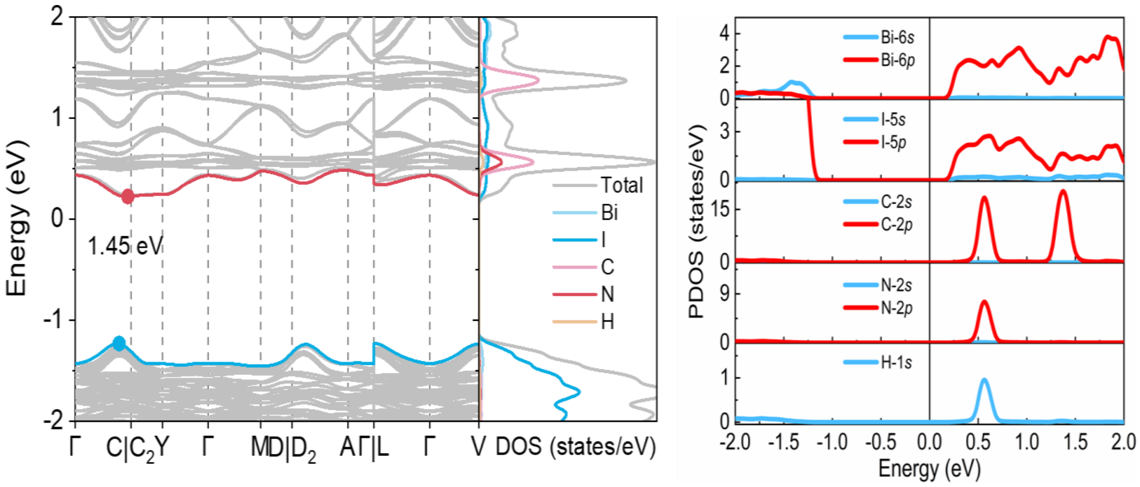

Supplementary Figure 9. Calculated band structure and Partial density of states spectra of **ABI** using PBE+SOC.

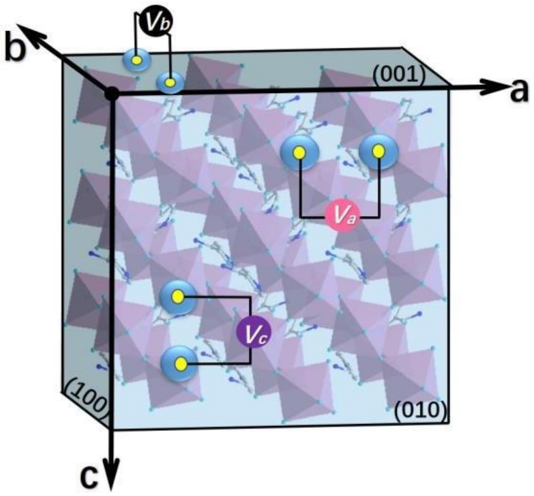

Supplementary Figure 10. Schematic diagram for photovoltages measurements. V_a, V_b, V_c refers to the photovoltages measured along the directions *a*, *b* and *c*, respectively.

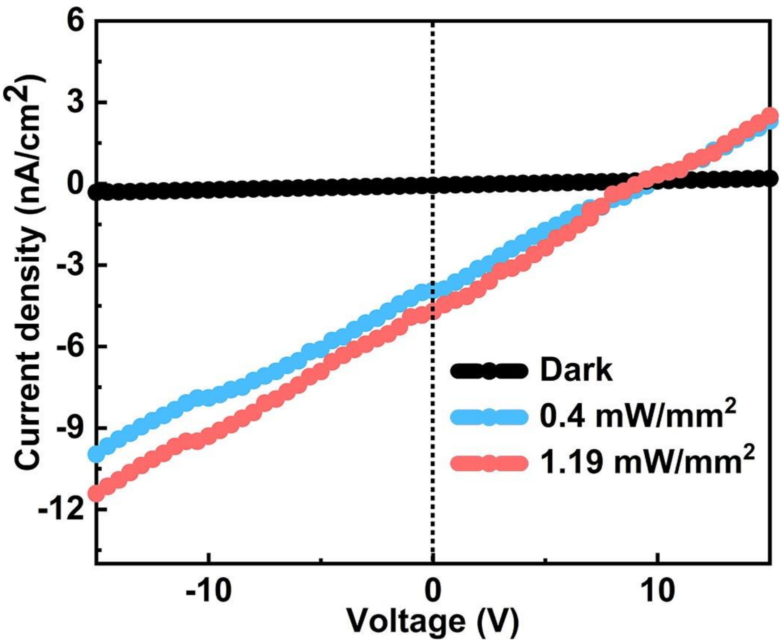

Supplementary Figure 11. I–V curves of the **ABI** measured in the dark and under different illumination intensities at 266 nm.

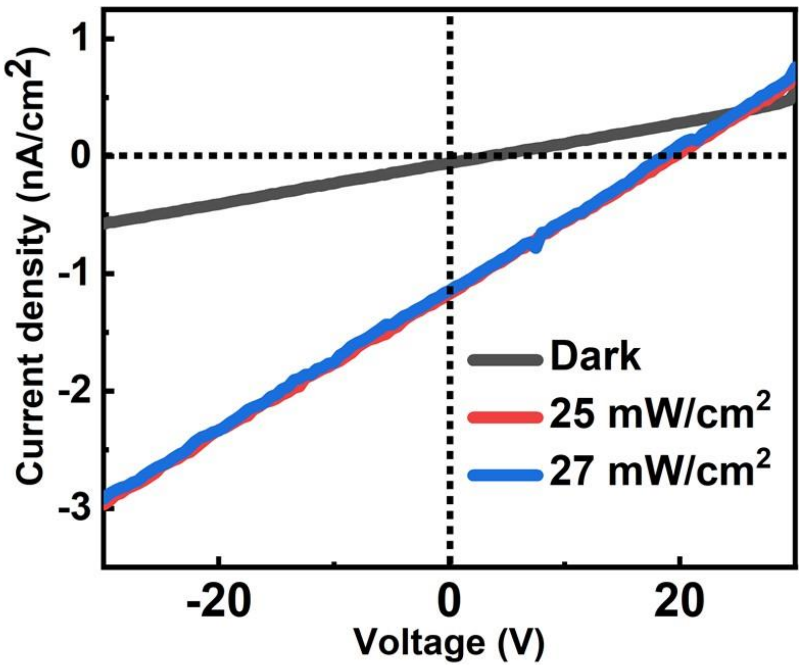

Supplementary Figure 12. I–V curves of the **ABI** measured in the dark and under different illumination intensities at 377 nm.

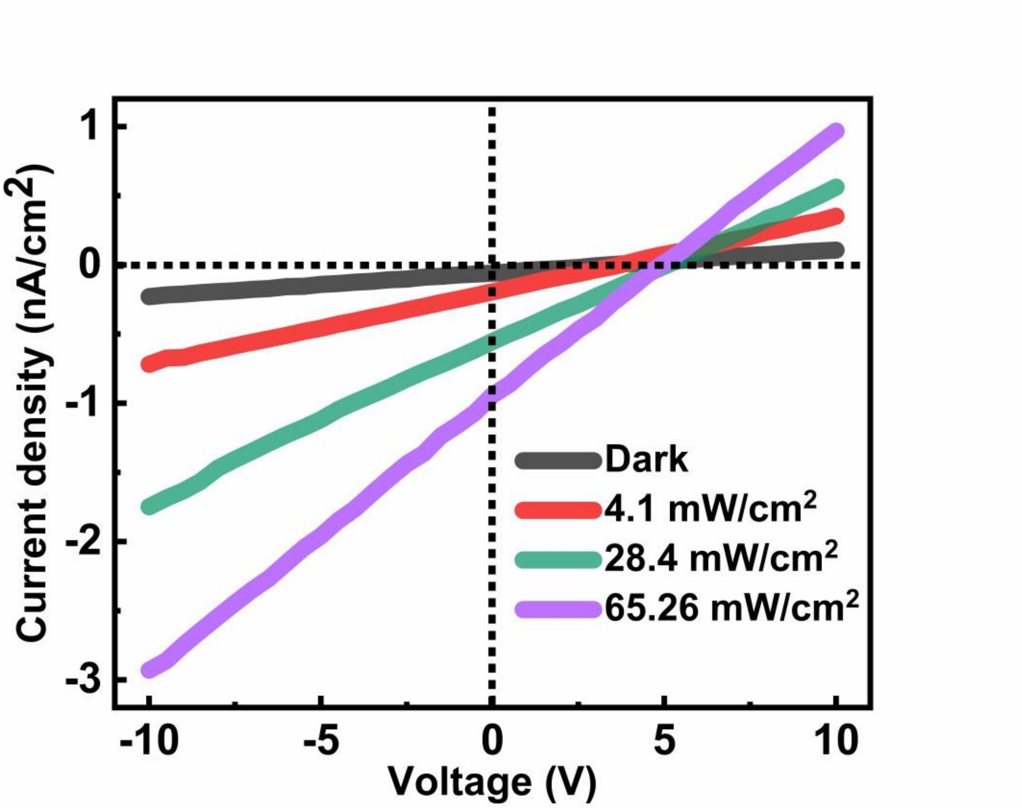

Supplementary Figure 13. I–V curves of the **ABI** measured in the dark

and under different illumination intensities at 520 nm.

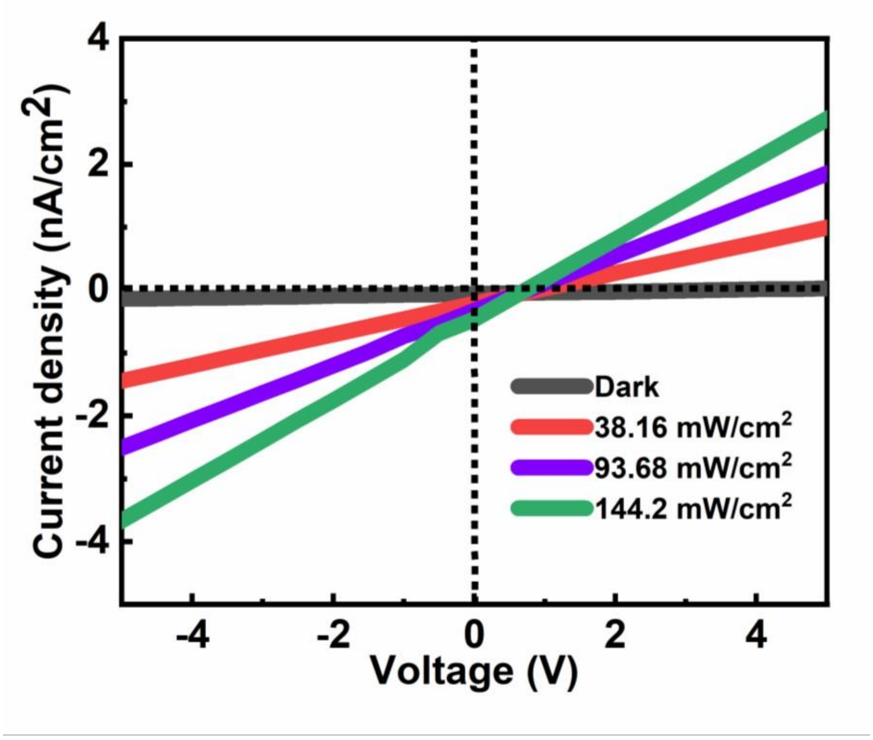

Supplementary Figure 14. I–V curves of the **ABI** measured in the dark and under different illumination intensities at 637 nm.

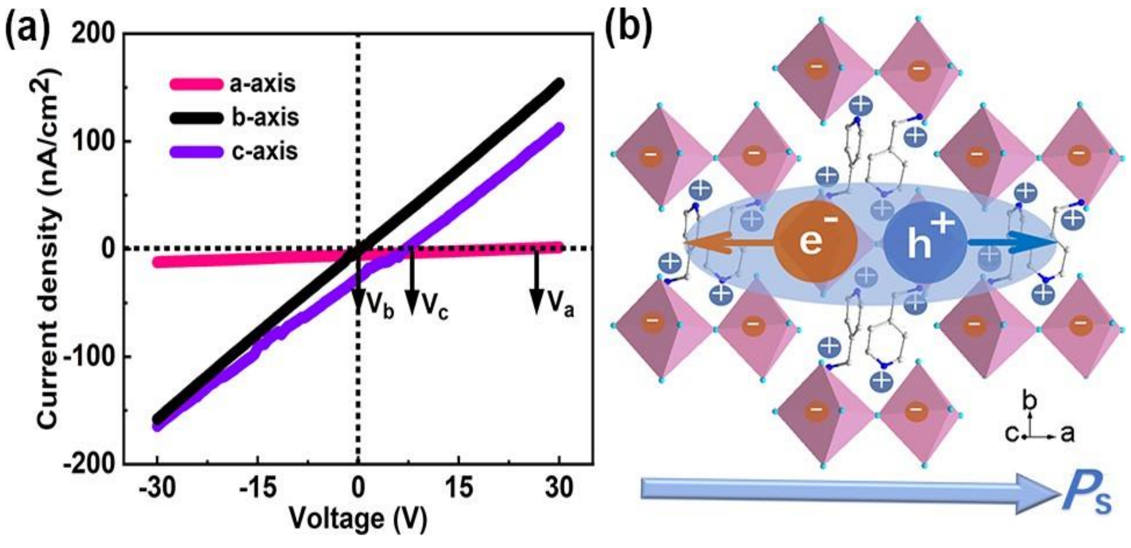

Supplementary Figure 15. Photovoltage dependence with polarization direction (a) *I*–*V* traces measured along the different axes. (b) Schematic illumination of photoexcited carrier separation under illumination.

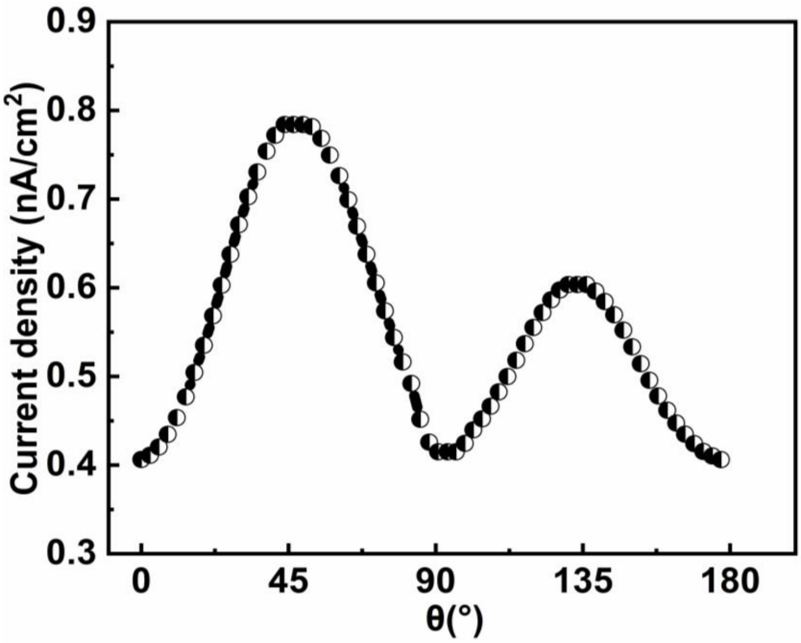

Supplementary Figure 16. Photocurrent measured as the function of rotation angle of λ/4 plate when the light is incident according the Figure 4a.

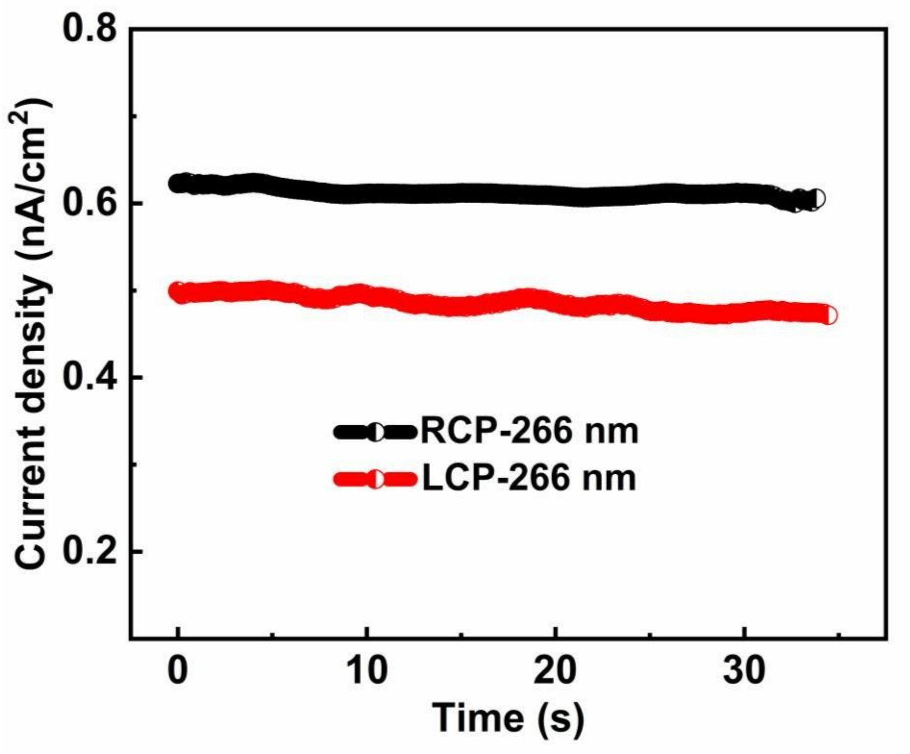

Supplementary Figure 17. The photocurrent density as a function of time under LCP-266 and RCP-266 nm irradiation recorded at V_{bias} = 0 V.

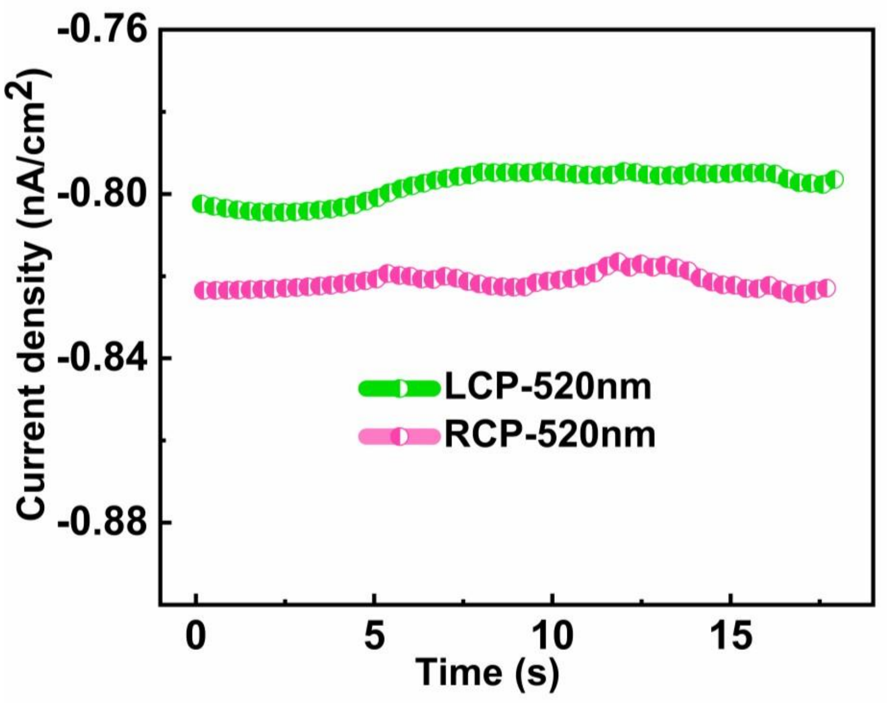

Supplementary Figure 18. The photocurrent density as a function of time under LCP-520 and RCP-520 nm irradiation recorded at V_{bias} = 0 V.

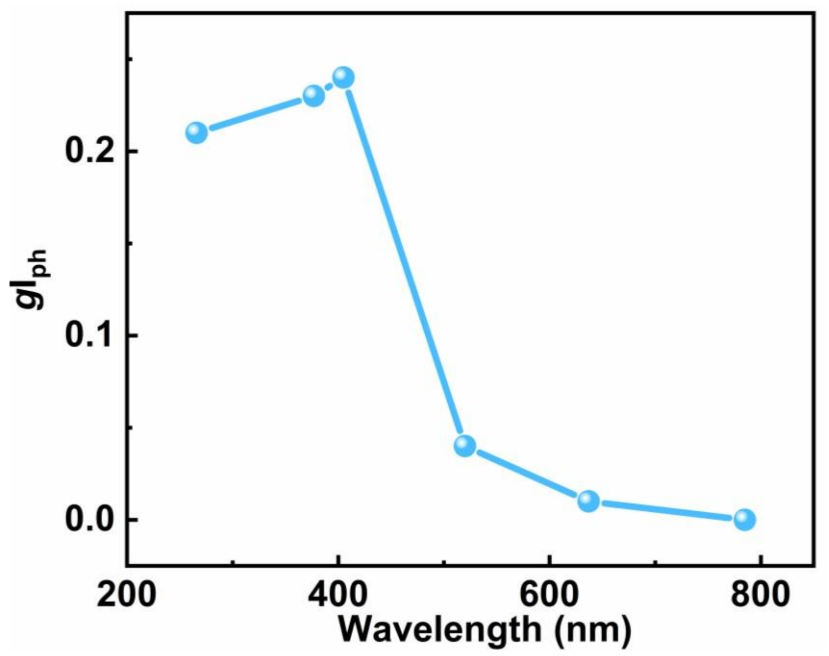

Supplementary Figure 19. g_{Iph} as a function of illumination wavelength at the zero bias.

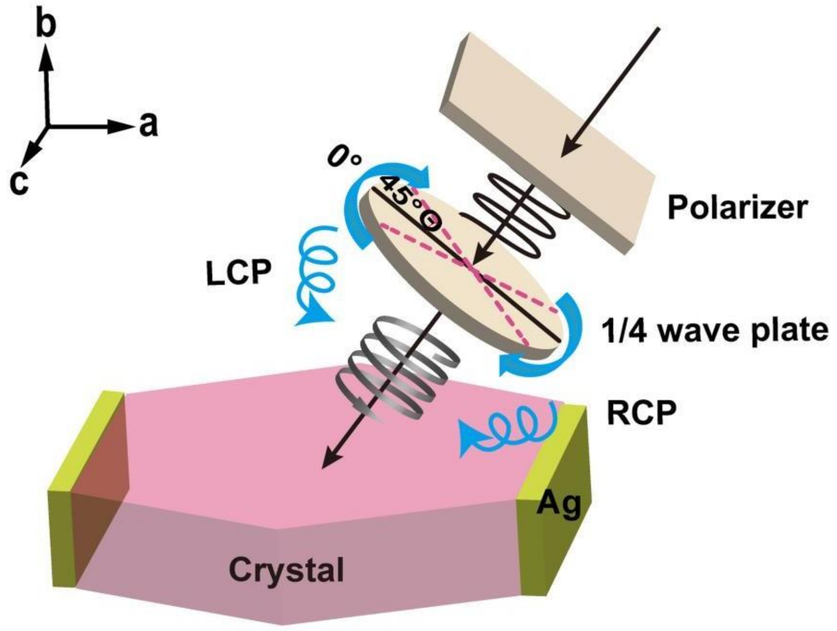

Supplementary Figure 20. The schematic view of the testing the CBPE device when the direction of incident light is reversed to that of Figure 4a.

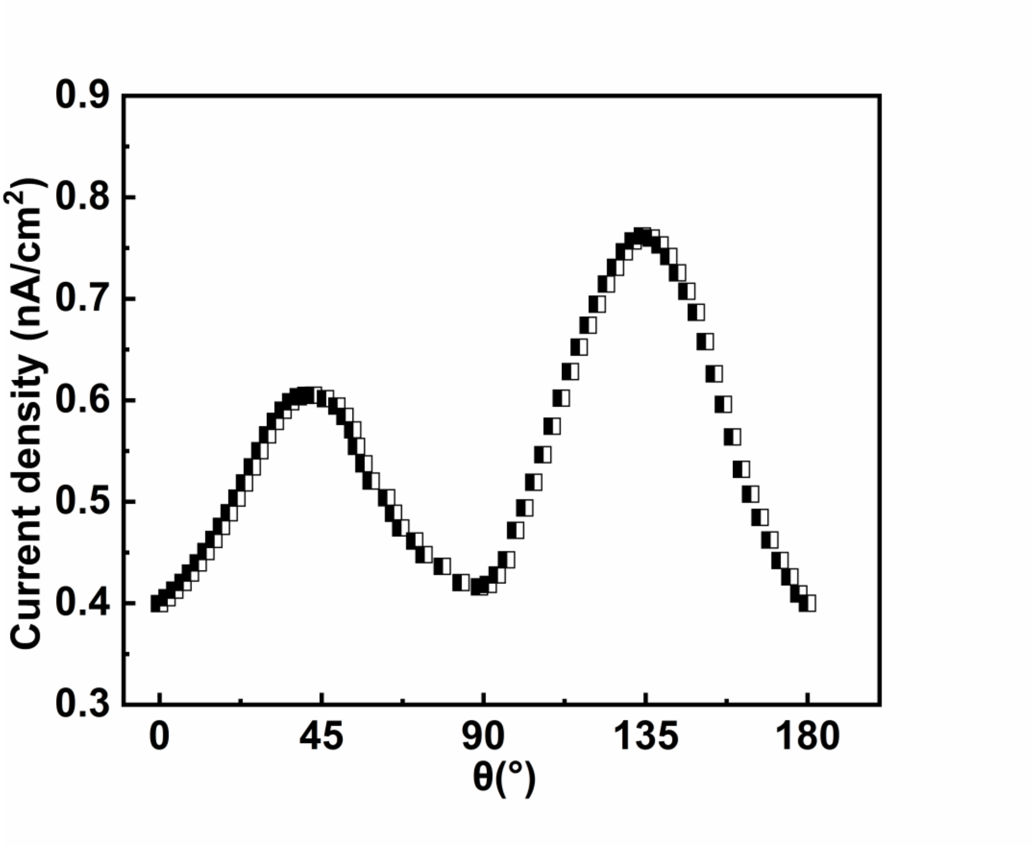

Supplementary Figure 21. Photocurrent measured as the function of rotation angle of λ/4 plate when the light is incident according to the Supplementary Table 5.

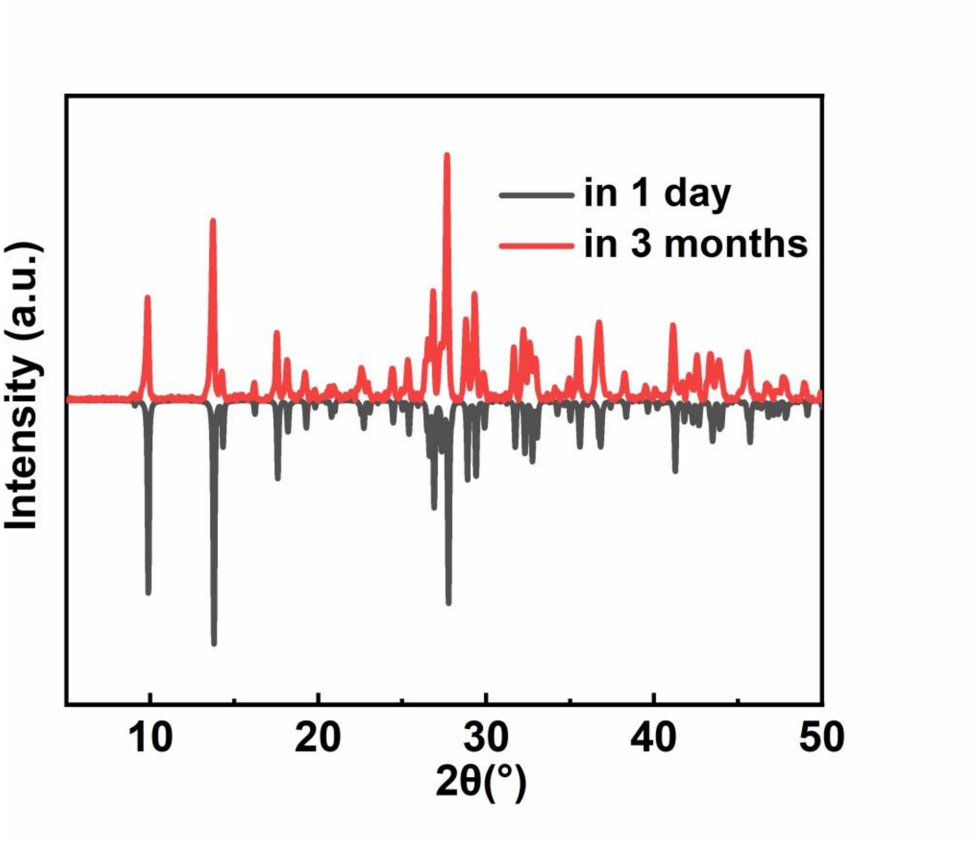

Supplementary Figure 22. The ambient stability of **ABI** XRD patterns.

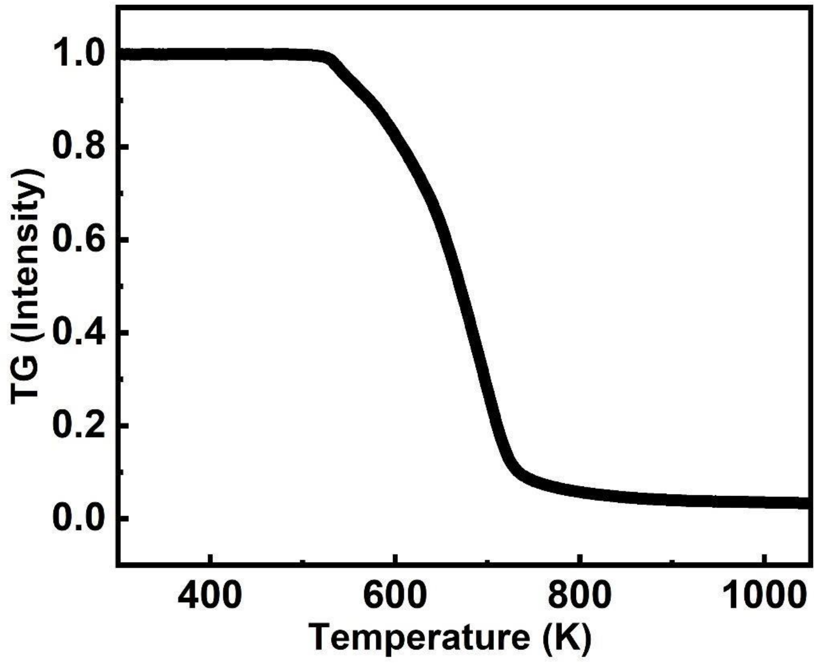

Supplementary Figure 23. Thermogravimetric analysis curve for **ABI**.

The result shows that **ABI** has a high thermal stability up to 523 K

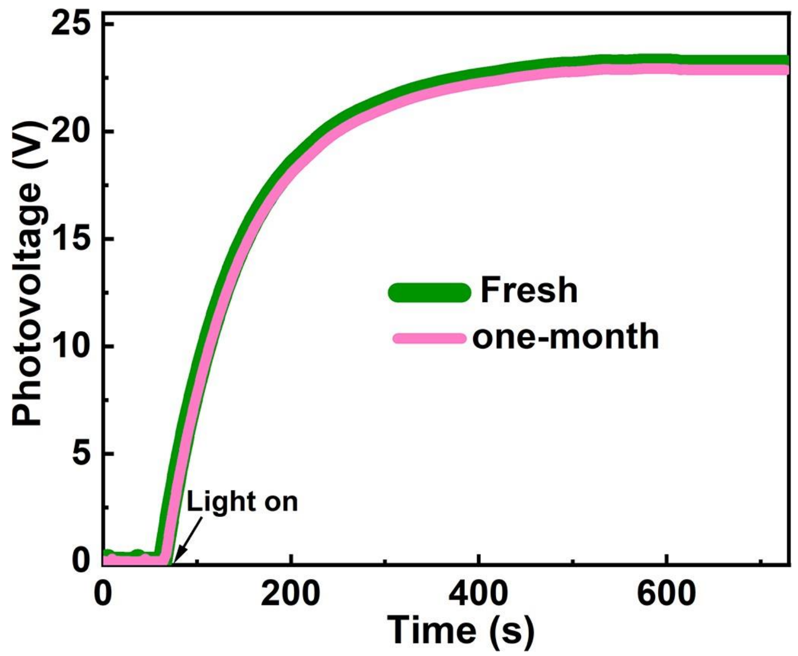

Supplementary Figure 24. The photovoltage stability.

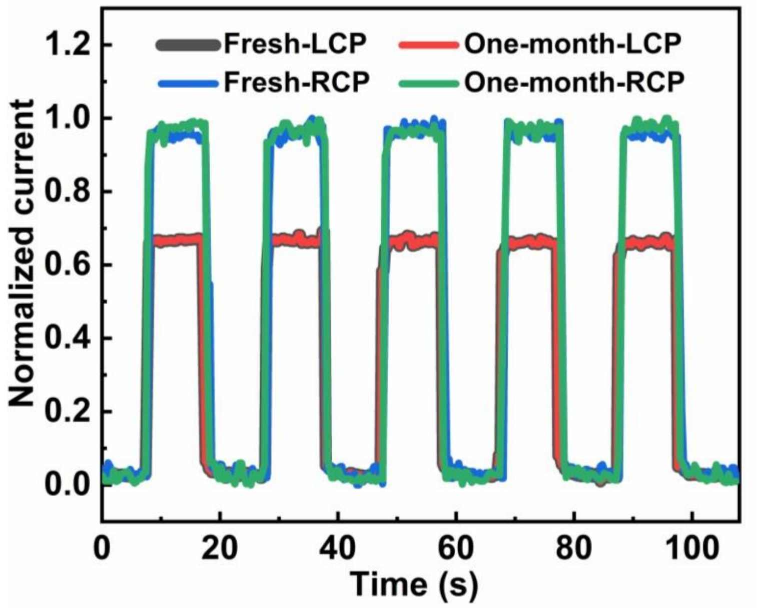

Supplementary Figure 25. Photocurrent of fresh and one-month aged **ABI** under LCP and RCP 405 nm light.

Supplementary Table 1. Crystal data and structure refinement for

compound ABI.

Name	**ABI**
Temperature/K	209.15
Empirical formula	$C_6H_{1I0}N_2BiI_5$
Crystal system	Monoclinic, Cc
Cell parameters	*a* = 19.492(2) Å<br>*b*=10.0829 (8) Å<br>*c*=17.081I3 (12) Å
V (Å^3)	3357.1(5)
Z, *ρ*cal. (g/cm^3)	8, 3.774
F(000)	3264.0
Theta range (°)	4.77-55.004
Limiting indices	- 25≤ h ≤ 24,<br>-12 ≤ k ≤ 13,<br>-22≤ l ≤ 22
Data/restraints/parameter	7635/98/256
Completeness	100
GOF	0.935
Final *R* indices [*I* > 2σ(*I*)]	R_1 = 0.0443,<br>wR_2 = 0.0770
R indices (all data)	R_1 = 0.0641,<br>wR_2 = 0.0868
Largest diff. peak and hole	1.40, -1.22 e.A^{-3}

Notes: The explainations of the B-level alerts in the CheckCIF routine of **ABI**: about the angles be 90 for a monoclinic cell. Anomalous photovoltaic effect only exists in the direction parallel to the *ac* plane, and is almost negligible in the direction of *b*-axis. Further, CBPE effect are detectable in the direction parallel to the *ac* plane. This phenomenon is consistent with the theoretical analysis of the chiroptical activity of space group *Cc*. Above experimental results can support the correctness of structural crystallization in *Cc* space group.

Supplementary Table 2. Selected Bi-I bond lengths for **ABI**

**ABI-1**	Bond lengths
Bi(1)-I(1)	3.235(5)
Bi(1)-I(2)	3.162 (3)
Bi(1)-I(3)	2.938 (5)
Bi(1)-I(4)	2.965 (5)
Bi(1)-I(5)	2.979 (3)
Bi(1)-I(6)	3.251 (5)
Bi(2)-I(1)	3.252(5)
Bi(2)-I(6)^{1}	3.235 (5)
Bi(2)-I(7)	3.040 (4)
Bi(2)-I(8)	2.959 (5)
Bi(2)-I(9)	2.948 (5)
Bi(2)-I(10)	3.079 (4)

Supplementary Table 3. Selected I-Bi-I bond angles for **ABI**

**ABI-1**	Bond angles
I (1I)-Bi(1)-I(6)	84.59 (17)
I (2)-Bi(1)-I(1)	85.42 (10)
I (2)-Bi(1)-I(6)	91.17 (10)
I (3)-Bi(1)-I(1)	87.81(12)
I (3)-Bi(1)-I(2)	87.58(11)
I (3)-Bi(1)-I(4)	98.15(15)
I (3)-Bi(1)-I(5)	91.13 (12)
I (3)-Bi(1)-I(6)	172.37(16)
I (4)-Bi(1)-I(1)	170.83 (11)
I (4)-Bi(1)-I(2)	87.87(10)
I (4)-Bi(1)-I(5)	95.21 (10)
I (4)-Bi(1)-I(6)	89.31 (12)
I (5)-Bi(1)-I(1)	91.61 (11)
I (5)-Bi(1)-I(2)	176.80 (15)
I (5)-Bi(1)-I(6)	89.73 (11)
I $(6)^1$-Bi(2)-I(1)	84.25(17)
I (7)-Bi(2)-I(1)	91.28 (11)
I (7)-Bi(2)-I$(6)^1$	90.99(11)
I (7)-Bi(2)-I(10)	177.08(15)
I (8)-Bi(2)-I(1)	89.92(12)
I (8)-Bi(2)-I$(6)^1$	174.02(16)
I (8)-Bi(2)-I(7)	90.47(12)
I (8)-Bi(2)-I(10)	92.25(12)
I (9)-Bi(2)-I(1)	174.64(16)
I (9)-Bi(2)-I$(6)^1$	87.51 (12)
I (9)-Bi(2)-I(7)	87.47 (11)
I (9)-Bi(2)-I(8)	98.35 (16)
I (9)-Bi(2)-I(10)	91.06 (11)
I (10)-Bi(2)-I(1)	89.81 (11)
I (10)-Bi(2)-I(1)	89.81 (11)

Supplementary Table 4. The asymmetry factor of various reported CPL detectors:

**compound**	**asymmetry factor**	**Ref.**
(*R*-α-PEA)PbI_3	0.1	[1]

[(R)- β -MPA]$_2$MAPb$_2$I$_7$	0.2	[2]
(R- α -PEA)$_2$PbI$_4$.	0.23	[3]
[(R)- β -MPA]$_4$AgBiI$_8$	0.3	[4]
(R-BPEA)$_2$PbI$_4$.	0.1	[5]
(R (or S)-NEA)PbI$_3$	1.73	[6]
[(R)-β-MPA]$_2$PbCl$_4$	0.1	[7]
(R,R) - and (S,S) - ProSQ - C6 Fullerene-blended BHJ photodiodes	0.1	[8]
**ABI**	0.24	***This work***

Calculation of polarization of **ABI** according to a point charge model

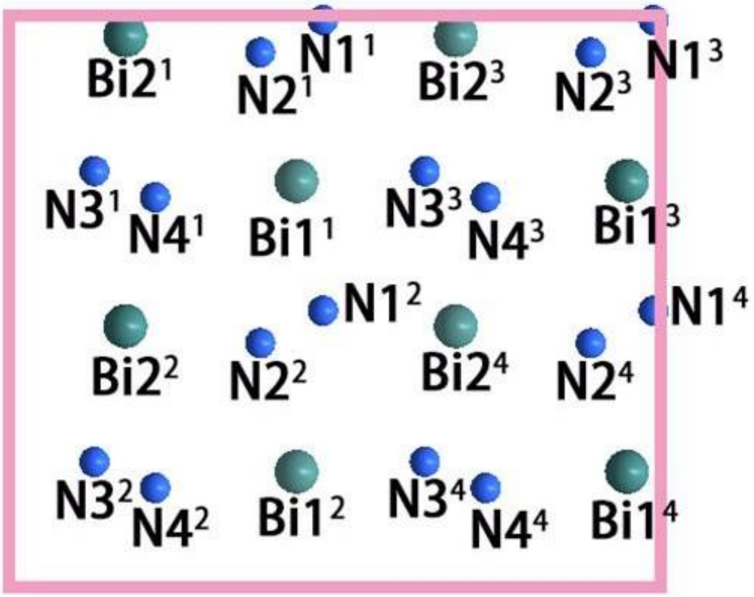

Supplementary Figure 26. Distribution of Bi and I atoms of **ABI** in a unit cell.

Based on the crystal structure data of **ABI**, we select a unit cell and assume that the centers of the positive charges of the $(4AMP)^{2+}$ and the

negative charges of the $(BiI_5)^{2-}$ are located on the N atoms and Bi atoms, respectively

Supplementary Table 5.

Atoms	Atoms coordinate		Coordinate of charge center
Bi1	Bi1[1](0.4419, 0.5083, 0.2949)	Bi1[2](0.441I9, 0.491I7, 0.7949)	(0.77523, 0.5, 0.5449)
	Bi1[3](0.9419,0.4917, 0.7949)	Bi1[4](0.9419,0.5083, 0.2949)	
Bi2	Bi2[1](0.1829, 0.4967, 0.0447)	Bi2[2](0.1829, 0.5033, 0.5447)	(0.34957, 0.5, 0.2947)
	Bi2[3](0.6829, 0.4967, 0.0447)	Bi2[4](0.6829, 0.5033, 0.5447)	
N1	N1[1](0.482, 0.781, 0.018)	N1[2](0.482, 0.219, 0.518)	(0.732,0.5,0.268)
	N1[3](0.982, 0.281, 0.018)	N1[4](0.982, 0.719, 0.518)	
N2	N2[1](0.387, 0.233, 0.074)	N2[2](0.387, 0.767, 0.574)	(0.637, 0.5, 0.324)
	N2[3](0.887, 0.733, 0.074)	N2[4](0.887, 0.267, 0.574)	
N3	N3[1](0.136, 0.717, 0.278)	N3[2](0.136, 0.283, 0.778)	(0.386,0.5,0.528)
	N3[3](0.636, 0.217, 0.278)	N3[4](0.636, 0.783, 0.778)	
N4	N4[1](0.228, 0.264,0.323)	N4[2](0.228,0.736,0.823)	(0.478,0.5,0.573)
	N4[3](0.728,0.764,0.323)	N4[4](0.728,0.236,0.823)	

The electric polarization (Ps) along a-axis can be estimated to be:
$Ps = \{[-8e \times (0.77523+0.34957)] + (0.732+0.637+0.386+0.478) \times 4e\} \times a / V$
$= -0.0664 \times 1.6 \times 10^{-19} \times 19.523 \times 10^{-10}$ C m/3379.51 $\times 10^{-30}$m^3)
$= -6.137 \times 10^{-3}$ C m^{-2}
$|Ps| = 6.137 \times 10^{-3}$ C m^{-2} = 0.6137 μC cm^{-2}
Thus, electric dipole moments μ = Ps V
$=2.074 \times 10^{-29}$ C·m = 6.22 1D
The electric polarization (Ps) along c-axis can be estimated to be:
$Ps = \{-8e \times (0.5449+0.2947) +(0.268+0.324+0.528+0.573) \times 4e\} \times c / V$
$=-0.0552 \times 1.6 \times 10^{-19} \times 17.1453$ x 10^{-10} C m/3379.51 $\times 10^{-30}$m^3
=-0.00448 C m^{-2}
|Ps| = 0.00448 C m^{-2} = 0.448 μC cm^{-2}
Thus, electric dipole moments μ = Ps V
$= 1.514 \times 10^{-29}$ C·m = 4.54 D

Supplementary References:

(1) Chen, C.*et al.* Circularly Polarized Light Detection Using Chiral Hybrid Perovskite. *Nat. Commun.* **10**, 1–7 (2019).

(2) Wang, L. *et al.* A Chiral Reduced-Dimension Perovskite for an Efficient Flexible Circularly Polarized Light Photodetector. *Angew. Chem. Int. Ed.* **59**, 6442–6450 **(2020)**.

(3) Wang, J. *et al. Spin-Dependent Photovoltaic and Photogalvanic Responses of Optoelectronic Devices Based on Chiral Two-Dimensional Hybrid Organic-Inorganic Perovskites. ACS Nano.* **15**, 588–595 **(2021)**.

(4) Li, D. *et al.* Chiral Lead-Free Hybrid Perovskites for Self-Powered Circularly Polarized Light Detection. *Angew. Chem. Int. Ed.* **60**, 8415–8418 (2021).

(5) Peng, Y. *et al.* Realization of Vis–NIR Dual-Modal Circularly Polarized Light Detection in Chiral Perovskite Bulk Crystals. *J. Am. Chem. Soc.* **143**, 14077–14082 (2021).

(6) Ishii, A. & Miyasaka, T. Direct Detection of Circular Polarized Light in Helical 1D Perovskite-Based Photodiode. *Sci. Adv.* **6**, 1–7 (2020).

(7) Zhang, X. *et al.* Heterogeneous Integration of Chiral Lead–Chloride Perovskite Crystals with Si Wafer for Boosted Circularly Polarized Light Detection in Solar-Blind Ultraviolet Region. *Small*, **17**, 2102884 (2021).

(8) Schulz, M. *et al.* Chiral Excitonic Organic Photodiodes for Direct Detection of Circular Polarized Light. *Adv. Funct. Mater.* **29**, 1900684 (2019).
